# Quantitative Mapping of Fibrotic Tissue Mechanics via Brillouin Spectroscopy

**DOI:** 10.1002/jbio.202500489

**Published:** 2025-11-14

**Authors:** Vsevolod Cheburkanov, Sujeong Jung, Mikhail Y. Berezin, Vladislav V. Yakovlev

**Affiliations:** ^1^ Department of Biomedical Engineering Texas A&M University College Station Texas USA; ^2^ Department of Radiology Washington University School of Medicine St. Louis Missouri USA; ^3^ The Institute of Materials Science & Engineering Washington University St. Louis Missouri USA; ^4^ Department of Physics and Astronomy Texas A&M University College Station Texas USA

**Keywords:** Brillouin spectroscopy, confocal microscopy, fibrosis, in situ imaging, tissue elasticity

## Abstract

Fibrosis is a pathological scarring process that disrupts tissue architecture, and is characterized by excessive extracellular matrix (ECM) deposition, leading to tissue stiffening and impaired organ function. Accurate quantification and spatial mapping of fibrotic tissue mechanics are critical for diagnosis, monitoring disease progression, and evaluating therapeutic responses. Here, we employ Brillouin microspectroscopy, a non‐invasive, label‐free optical technique, to quantify the mechanical properties of human fibrotic tissue in in situ. We show that Brillouin spectroscopy distinguishes fibrotic tissue from healthy tissue on the basis of localized differences in the complex longitudinal modulus and enables real‐time monitoring of dynamic alterations in viscoelastic properties during fibrogenesis. To our knowledge, this is the first demonstration of Brillouin spectroscopy for in situ characterization of fibrosis and wound healing in a human model. These findings underscore Brillouin microspectroscopy's potential application as a promising diagnostic and monitoring tool for fibrotic diseases.

## Graphical abstract

Brillouin spectroscopy distinguishes fibrotic from healthy tissue based on localized stiffness changes and enables real‐time monitoring of dynamic alterations in viscoelastic properties during fibrogenesis.

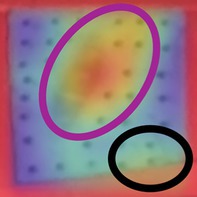

## 1 Introduction

Fibrosis is a pathological process characterized by the excessive production and deposition of extracellular matrix (ECM) components—primarily collagen—in response to tissue injury or chronic inflammation. This disruption in the normal balance between ECM synthesis and degradation leads to tissue stiffening, scarring, and impaired function [1]. Over time, the accumulation of fibrotic tissue can hinder organ repair, reduce blood flow, and compromise overall tissue functionality.

Fibrosis can affect multiple organs, including the heart, lungs, liver, kidneys, skin, peripheral nerves [2], and bone marrow [3, 4]. Its etiology is diverse, encompassing chronic inflammatory diseases, persistent infections, autoimmune conditions, allergic responses, chemical insults (e.g., chemotherapy or radiation), and physical injury. For example, chronic hepatitis can lead to liver fibrosis, ischemia and hypertension are major contributors to cardiac fibrosis [3], and trauma is a common cause of skin fibrosis [5].

Despite organ‐specific variations, the underlying mechanisms of fibrosis share common features [6], notably the transformation of fibroblasts into myofibroblasts, which secrete large amounts of ECM. Additionally, immune and endothelial cells, along with a complex network of cytokines and growth factors, drive the fibrotic cascade. While understanding these biological processes is critical for developing targeted antifibrotic therapies, equally important is the ability to visualize and monitor fibrosis, particularly in its early stages.

Current diagnostic approaches include tissue biopsy, immunohistochemistry (IHC) [7, 8], magnetic resonance imaging (MRI) [9, 10], ultrasound elastography [11, 12], optical coherence tomography (OCT) [13, 14, 15], laser speckle imaging [16, 17], blood‐based biomarkers [18, 19], and functional assays. These tools span from molecular and histological assessments to advanced non‐invasive imaging modalities [9, 20]. Clinically, imaging techniques like MRI, CT, and ultrasound are routinely used for staging fibrosis and guiding treatment. However, while biopsies remain the gold standard, they are invasive and subject to sampling errors and inter‐observer variability [21]. Conversely, standard imaging methods lack sensitivity for early‐stage disease and cannot differentiate between active fibrogenesis and stable scarring [22].

Addressing the clinical need for better imaging tests for fibrosis, several fibrosis‐specific methods have been developed. Given the major difference between fibrotic tissue and healthy tissue lies in the difference in elasticity, vibration‐controlled transient elastography [23], strain wave elastography (SWE) [20] and magnetic resonance elastography (MRE) [24] have been utilized. In these techniques, low‐frequency vibrations are generated and the propagation of shear waves is detected to create electrograms that map tissue stiffness. However, most of these techniques have poor spatial resolution without mapping viscoelastic properties of the tissue at the microscopic level [25, 26].

Brillouin microspectroscopy presents a promising alternative. This label‐free, non‐destructive optical technique measures tissue viscoelasticity with high spatial resolution using a confocal geometry. Spontaneous Brillouin scattering has been used to generate high‐resolution mechanical maps of biological tissues [27, 28, 29, 30] and has found applications in the characterization of biomedical materials on a microscopic scale [31, 32]. This imaging technique has been used to study the changes in mechanical properties during fibrosis progression in murine bladder tissue [33].

Despite its potential, Brillouin microscopy has been underutilized in imaging thick (> 1 mm), heterogeneous, and highly scattering tissues typical of fibrosis. Previous studies, while impactful, have largely been limited to transparent, such as lens [27] and zebrafish [29] or excised samples, such as tendon [34] and bladder lining [33]. To our knowledge, the application of Brillouin spectroscopy to a dermal fibrosis model in a human in situ setting has not been reported.

To address this gap in knowledge, we have developed a novel Brillouin imaging system that incorporates a molecular absorber, capable of suppressing elastic scattering by over six orders of magnitude [28, 35, 36, 37]. These foundational studies set the stage for the current investigation.

In this study, as a proof‐of‐principle we applied this advanced Brillouin imaging platform to assess human fibrotic tissue from the upper extremity, opportunistically obtained during routine sample collection. Although gross examination suggested normal healing, Brillouin imaging revealed extensive fibrotic remodeling at the site of injury. To further enhance the impact of the study and validate Brillouin spectroscopy as a reliable tool for quantitative fibrosis analysis, a more rigorous investigation is needed, incorporating multiple replicates and complementary control methods such as tissue dissection followed by histological analysis.

The participant was informed about the study's objectives and procedures, and was familiarized with the imaging modality, including its potential benefits and risks. Based on this information, the participant provided voluntary and informed consent to participate in the study and to have the results published.

## 2 Materials and Methods

### 2.1 Sample Description and Preparation

**FIGURE 1:**
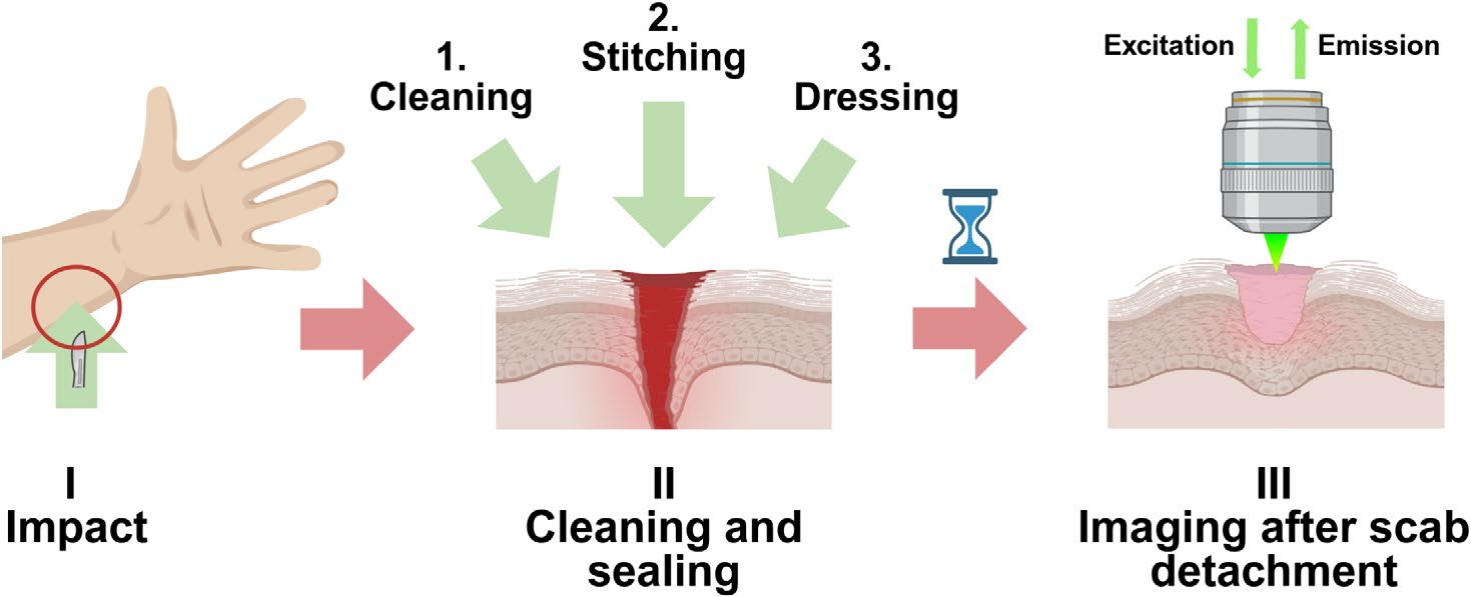
Schematic representation of the process of injury occurrence, treatment, and imaging. (I) Dorsal side of the left forearm was accidentally damaged with a forceful impact on a blunt stamped steel object with the radius of curvature of approximately 3 mm, approximately 50 mm up from the wrist joint. (II) The area of epithelium damage was cleaned with clean water. Edges of the wound were forced together and topical skin adhesive was applied to maintain wound closure. Clean dressing was applied to provide rigidity to the closure. (III) After 3 weeks the injure site was imaged with the Brillouin microspectrometer. Figure created with BioRender.com.

A sample of epidermal fibrotic tissue was obtained from a human following a recovery of a dermal injury to the left forearm on the dorsal side, induced by an impact with a blunt object. Data acquisition flow from the onset of injury to the imaging is shown in Figure 1.

After the wound was promptly cleaned and dressed, standard procedures for wound closure were implemented. The open wound was sealed using topical skin adhesive, secured with tape to minimize the gap between the rupture edges, and covered with sterile gauze and a bandage to prevent contamination.

**FIGURE 2:**
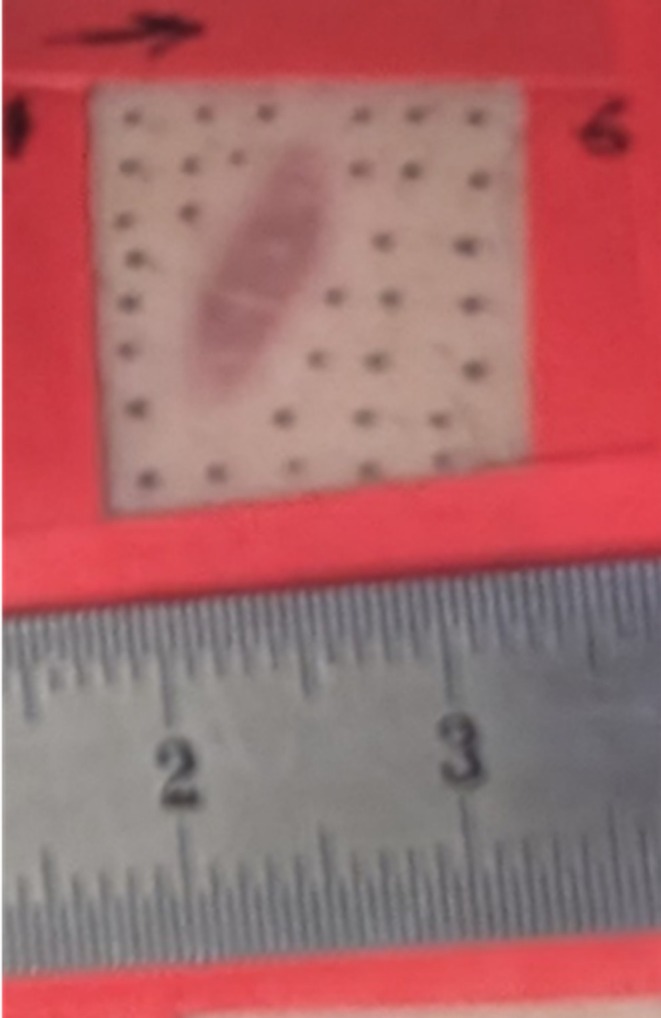
Photographic recording of prepared damage site with scale for reference.

One week post‐injury, no signs of inflammation were noticed, indicating the progression of normal wound healing. Two weeks post‐injury, the wound was cleaned of adhesive residue and bandaging, exposing the fibrotic tissue to the external environment. Three weeks post‐injury, a region of skin on the dorsal side of the left forearm, approximately 15 × 15 mm^2^ in size (Figure 2), was highlighted with labeling tape and the exposed part of the dermis with the injure site was imaged using the Brillouin microspectrometer [35, 38].

To prevent laser‐induced tissue damage and minimize temperature‐induced artifacts, the interrogation points were spaced adequately apart. The imaged area was shaved, leaving the hair follicles and approximately 500 μm of hair above the epidermis. Data collected from remaining hair shafts were automatically removed by a median filter during processing.

### 2.2 Setup Description

**FIGURE 3:**
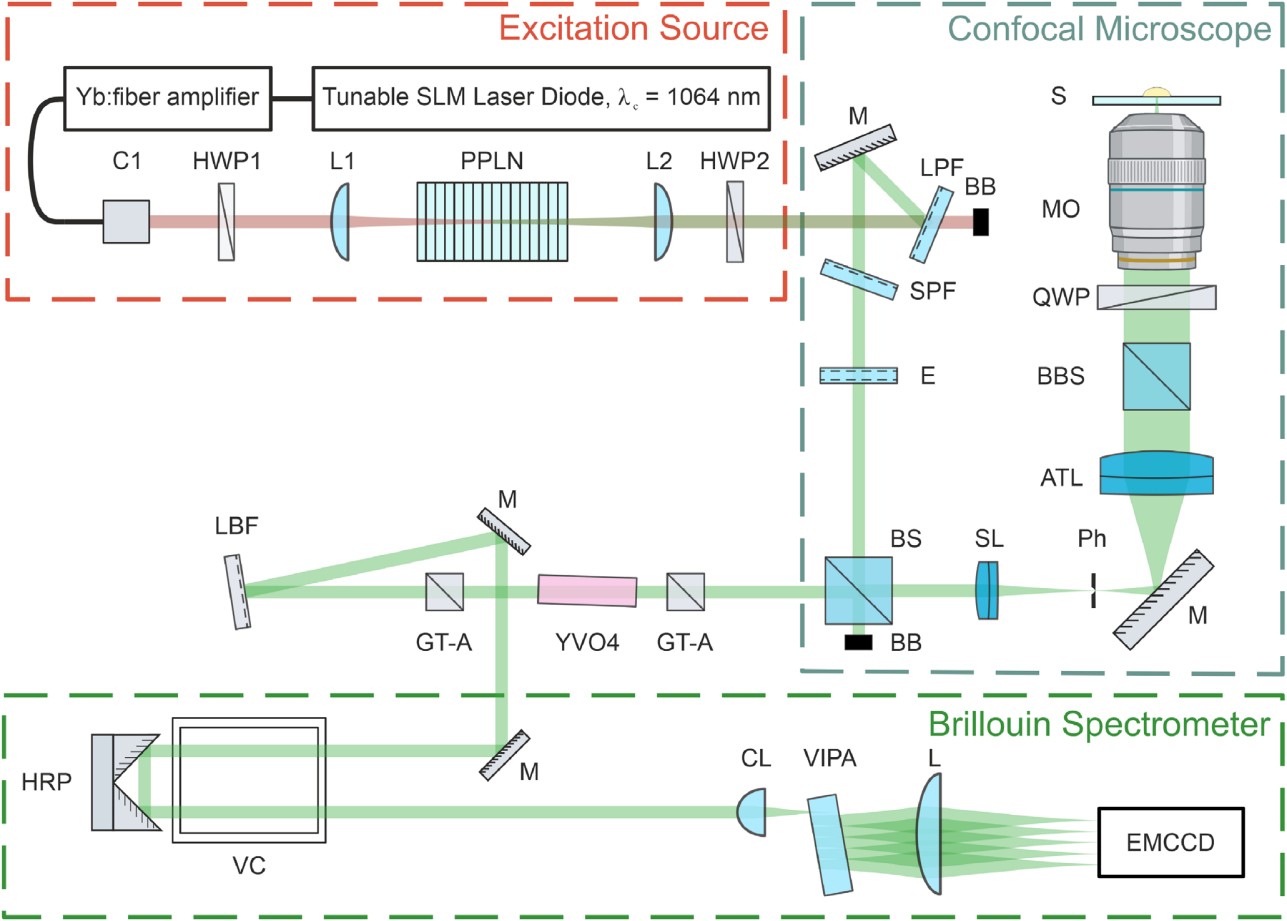
Brillouin confocal microspectrometer layout. C—fiber collimator, HWP1–1064 nm half‐wave plate, L—plano‐convex lens, PPLN—periodically poled lithium niobate second harmonic generation crystal, HWP2–532 nm half‐wave plate, BB—beam block, LPF—long‐pass filter, M—mirror with dielectric reflective coating, SPF—short‐pass filter, E—Fabry‐Perot etalon, BS—polarizing beamsplitter cube, SL—achromatic doublet scan lens, Ph—precision pinhole, ATL—achromatic tube lens (200 mm, focal length), BBS—broadband beamsplitter in quick‐insert mount, QWP—532 nm quarter‐wave plates, MO—microscope objective lens, S—sample, GT‐A—Glan‐Taylor calcite polarizer, YVO4—Yttrium Orthovanadate crystal, LBF—532 nm Bragg grating notch filter, HRP—hollow‐roof prism, VC—Iodine vapor cell, CL—cylindrical lens, VIPA—Virtually Imaged Phase Array, EMCCD—CCD with electron multiplying amplification capabilities.

The schematic of this setup is shown in Figure 3. The system includes an excitation source, a confocal microscope with a double‐pass confocal pinhole, and the home‐built single‐stage Virtually Imaged Phase Array (VIPA) Brillouin spectrometer.

A custom‐built 532 nm laser with a linewidth of less than 1 MHz was utilized as the excitation source. This wavelength was produced as the second harmonic of 1064 nm laser radiation within a periodically poled $\mathrm{MgO}:{\text{LiNbO}}_{3}$ crystal (Covesion Ltd.). The 1064 nm light was generated by a tunable single longitudinal mode laser diode (Koheras Adjustik Y10, NKT Photonics) and subsequently amplified using an Yb‐doped fiber amplifier (Koheras Boostik HPA Y10, NKT Photonics).

The desired wavelength of 532 nm was effectively isolated from the residual 1064 nm pump light through the use of a combination of long‐pass (LPF) and short‐pass (SPF) filters. The authors have validated the spectral line of the emitted radiation possessing the FWHM of < 100 MHz. To further clean the spectra of undesirable spectral components a fused silica Fabry‐Perot etalon (Lightmachinery Inc.) with free spectral range (FSR) of 29.98 GHz was used.

The microscope body was designed and constructed using standard optomechanical components (Thorlabs). The laser power delivered to the sample was regulated through the use of a 532 nm half‐waveplate in conjunction with a polarizing beamsplitter cube (BS, Thorlabs PBS251). A polymer quarter‐waveplate (QWP) was employed to achieve orthogonal polarization between the incident and scattered light, transmitting the scattered light through the BS towards the Brillouin spectrometer for detection.

To ensure optimal beam expansion, the laser beam was enlarged to a diameter of 10 mm at $1/e^{2}$ using a Keplerian telescope beam expander. A precision pinhole was positioned at the intermediate image plane of the telescope, serving to clean up the excitation beam and provide spatial filtration of the collected signal. The microscope was designed to be compatible with the Nikon CFI60 objectives, utilizing a tube lens (ATL) with a focal length of 200 mm. The microscope objective (MO) delivered approximately 10 ± 0.3 mW of 532 nm radiation to the sample while simultaneously collecting the scattered photons.

The signal was acquired utilizing a 20X Nikon Plan Fluorite dry objective with a numerical aperture (NA) of 0.5. This objective lens provided an optimal balance between axial resolution (< 5 μm), imaging depth (> 50 μm), signal collection efficiency, and a long working distance (2 mm), facilitating effective imaging of the fibrotic site.

The confocal pinhole was selected to transmit no more than 1 Airy Unit (AU), thereby leveraging the advantages of confocal microscopy, including optical sectioning of the sample and isolation of data from individual layers.

Fluorescence from the sample was separated using a 532 nm Bragg grating notch filter (LBP) with the rejection ratio of ≈30 dB and the FWHM of 4 cm^−1^. A 532 nm laser line filter (LLF) was also installed at the entrance pupil of the Brillouin spectrometer.

To further suppress the signal from elastically scattered photons a polarization rotation filter with FSR of 15 GHz was utilized. It comprised of a Glan‐Taylor (GT‐A) polarizer and analyzer pair. With the polarization rotation is achieved with a pair of a‐cut ${\mathrm{YVO}}_{4}$ crystals with the total length of 30 nm. Effective aperture of the crystal was 5 × 5 mm^2^ (Conex Optics Inc.).

The collected and filtered signal was coupled into the entrance pupil of a custom‐designed Brillouin spectrometer. To mitigate the influence of elastically scattered photons, an iodine vapor cell (Thorlabs) was employed, maintained at a temperature of 70°C. By implementing a double‐pass beam propagation configuration and precisely tuning the laser output wavelength to coincide with the strongest absorption band of molecular iodine, suppression ratios exceeding 60 dB were achieved. The optimal absorption wavelength was determined to be 531.94 nm, corresponding to line #638 [39], with a wavenumber of 18,801.244 cm^−1^.

The Brillouin scattering signal was analyzed using a high‐dispersion, custom‐built single‐stage VIPA spectrometer. The VIPA (OP‐6721‐3371‐2, Lightmachinery Inc.) was specifically optimized for 532 nm and featured a FSR of 29.98 GHz (1 cm^−1^). Signal spectra were recorded with a water‐cooled EMCCD camera (Andor Newton 970P, Oxford Instruments).

### 2.3 Field of View Settings and Signal Processing

Control of the imaging depth was achieved using a manual linear stage with a micrometer screw, moving the MO along its optical axis. After positioning the sample on the microscope stage, the laser waist was moved axially toward the tissue surface. A noticeable increase in the elastically scattered photon signal indicated proximity to the surface. The signal was then maximized, and the corresponding micrometer reading was recorded as the reference point. Subsequent imaging depths were measured relative to this value.

Acetone was used as a standard sample for spectrometer calibration. The collected Brillouin spectra of acetone were used to generate a calibration curve, which allowed the conversion of the spectral line positions on the EMCCD sensor to the Brillouin frequency shift domain.

The spectral analysis algorithm is described in detail elsewhere [38].

## 3 Results and Discussion

**FIGURE 4:**
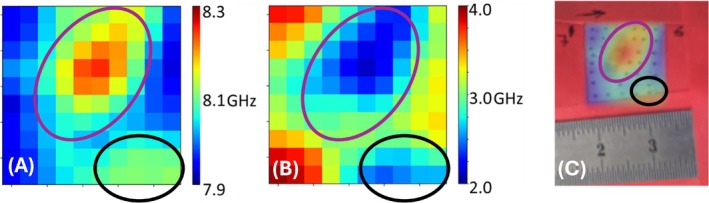
Spatial map of Brillouin shift and FWHM values corresponding to picture of the sample. (A) Spatial distribution of the Brillouin shift values within the investigated region. (B) Spatial distribution of the Brillouin line FWHM values within the investigated region. (C) Brillouin shift map overlaid with the image of the scar site. Purple ellipse highlights the scar within the field of view. Black ellipse highlights the region of skin that was pinched by the labeling tape.

Using the experimental protocol and the data analysis algorithm described above, maps of Brillouin frequency shift and Brillouin spectral line FWHM values were generated. The Brillouin frequency shift map was subsequently overlaid with the widefield image of the fibrotic wound and is presently shown in the rightmost part of the illustration Figure 4.

The purple elliptical region highlighted in Figure 4 represents the location of the scar.

**FIGURE 5:**
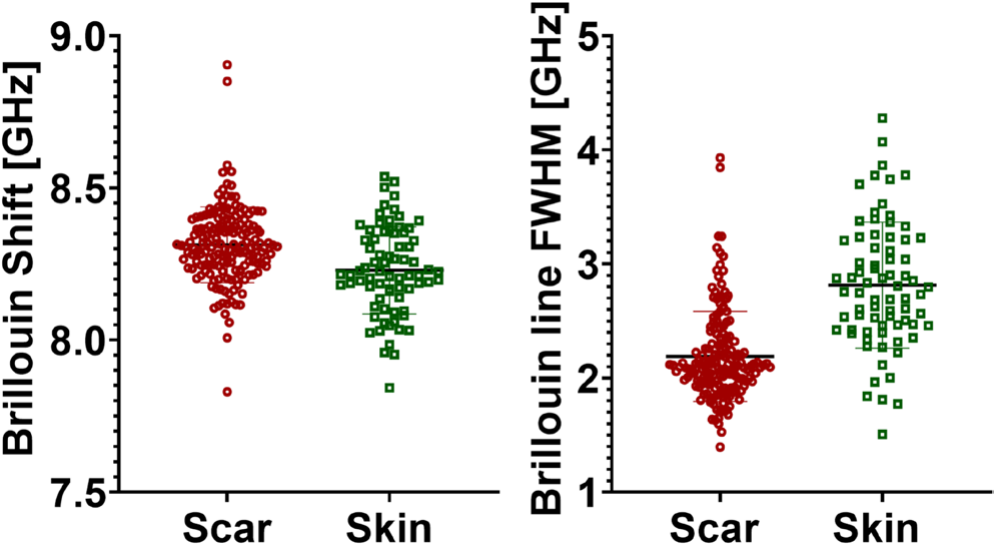
Distribution of Brillouin spectral line shift and FWHM values. (A) Statistical distribution of Brillouin shift values for the two distinct tissue regions (scar *N* = 200; skin *N* = 80). Brillouin shift values retrieved from the scar tissue are depicted as red circles, while values retrieved from the skin are shown as green squares. The difference in the average Brillouin shift value between the regions is significant (*p* < 0.0001, unpaired *t*‐test), and the mean values are 8.31 ± 0.15 GHz and 8.22 ± 0.15 GHz, respectively. (B) Statistical distribution of Brillouin FWHM values for the two distinct tissue regions. Brillouin FWHM values retrieved from the scar tissue are depicted as red circles, values retrieved from the skin are shown as green squares. The mean values are 2.19 ± 0.35 GHz for the scar region and 2.81 ± 0.55 GHz for the healthy skin. The difference in the average Brillouin shift value between the regions is significant (*p* < 0.0001, unpaired *t*‐test).

Data from within this region (purple ellipse, Figure 4), as well as from the adjacent tissue, were collected to accurately determine the mean values of the Brillouin line properties. Data from points within the region were compiled into a value distribution plot, shown in Figure 5. The same wound region was imaged multiple times, with the overall field‐of‐view shift not exceeding 500 μm. Consequently, each pixel in Figure 4 represents multiple spectra, ensuring independent sampling.

The presented data indicate that the Brillouin shift in the wound region is significantly higher compared to healthy skin, with mean values of 8.31 ± 0.15 GHz and 8.22 ± 0.15 GHz, respectively (*p* < 0.0001, unpaired *t*‐test). Median values of Brillouin shift retrieved from the regions are 8.31 and 8.22 GHz.

In contrast, the scar region exhibited smaller linewidth (calculated as FWHM) values relative to healthy skin, with mean values of 2.19 ± 0.35 GHz and 2.81 ± 0.55 GHz, respectively (*p <* 0.0001, unpaired *t*‐test). Median value of FWHM for the two regions is 2.10 and 2.81 GHz, respectively.

From these spectral properties, the localized mechanical characteristics can be inferred using the complex longitudinal modulus [40], which encodes information about the high‐frequency viscoelastic behavior of the material under investigation.

Considering that the real part of the longitudinal modulus ($M^{\prime }$) is proportional to the square of the Brillouin shift value, and using the mean values for the respective samples, we estimate that $M^{\prime }$ of the wound region is approximately 2% larger than that of the interrogated healthy skin. These results fall well within the sensitivity of the system, as previously reported [38]. Although the observed differences in the high‐frequency longitudinal modulus appear small, they correspond to substantial differences in the storage modulus measured using contemporary techniques [41]. This discrepancy is largely due to the fact that Brillouin spectroscopy specifically probes the high‐frequency component of the modulus, rather than the low‐frequency response.

The complex part of the longitudinal modulus (${M^{\prime }}^{\prime }$) is proportional to the product of the Brillouin shift and FWHM values, as described above. Based on this relationship and using mean values of Brillouin shift and Brillouin line FWHM values per region, we infer that the ${M^{\prime }}^{\prime }$ of the wound region is approximately 26% larger compared to healthy skin.

**FIGURE 6:**
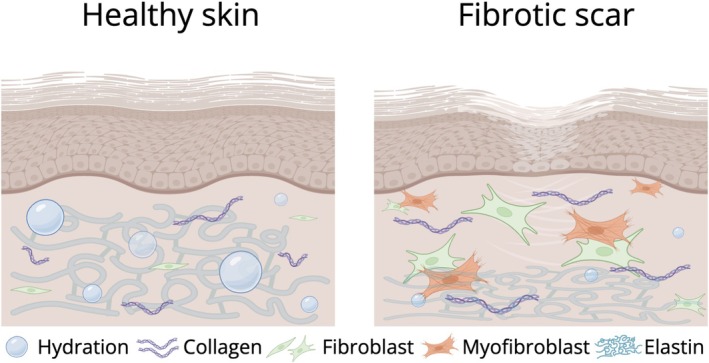
Comparison of healthy skin and fibrotic scar. Schematic illustration comparing the dermal architecture of (left) healthy skin and (right) fibrotic scar tissue. In healthy skin, the dermis contains loosely organized collagen fibers (purple), abundant elastin fibers (blue wavy lines), normal fibroblasts (green), and sufficient hydration (blue spheres), producing a compliant and viscoelastic environment. In fibrotic scar tissue, dense and aligned collagen bundles, numerous activated fibroblasts and myofibroblasts (orange), reduced elastin content, and diminished hydration contribute to a stiffer, less viscous microenvironment. This structural remodeling underlies the elevated Brillouin frequency shift and reduced linewidth observed in scar regions compared to healthy skin. Created with BioRender.com.

These results can be interpreted in the context of the characteristic architecture of fibrotic skin, as illustrated in Figure 6.

Dermal tissue possesses a layered organization, with epidermal thickness varying across different anatomical site [42, 43]. In the forearm examined here, histological data indicate that the epidermis is typically thinner than 100 μm [44].

Previous studies report that 532 nm laser light penetrates skin only to depths on the order of hundreds of micrometers, rather than millimeters [45, 46]. However, these results do not account for beam geometry, numerical aperture of the optics and the efficiency of backscattered photon collection. Our observations indicate a significant decrease in the collected Brillouin scattered signal, dropping by over an order of magnitude at 200 μm. At this depth, photons are highly susceptible to multiple scattering, which affects both the linewidth and the spectral peak position [47], thereby reducing the reliability of the data and the observed SNR. To mitigate this limitation of 532 nm laser excitation, the imaging depth is restricted to 100–125 μm, where multiple scattering effects are minimized and the SNR remains high.

This depth range is sufficient to access the dermis below the epidermis and to quantify the viscoelastic properties of both fibrotic and adjacent healthy regions.

Dermal tissues are highly viscoelastic and their mechanical response evolves during wound healing, and changes as they recover from injury [48, 49]. In uninjured skin, low‐load testing demonstrates a bidirectional response with a pronounced viscous component driven by interstitial fluid mobility [50]. In contrast, scar tissue exhibits increased stiffness at low loads together with altered fluid retention, consistent with dense, oriented collagen networks [51, 52, 53, 54, 55].

Our Brillouin measurements confirmed this trend: scars showed higher mean frequency shift ($\nu_{B,\text{scar}}=8.31\pm 0.15\;\mathrm{GHz}$ vs. $\nu_{B,\text{skin}}=8.22\pm 0.15\;\mathrm{GHz}$, p<0.0001) and lower linewidth (${\text{FWHM}}_{\text{scar}}=2.19\pm 0.35\;\mathrm{GHz}$ vs. ${\text{FWHM}}_{\text{skin}}=2.81\pm 0.55\;\mathrm{GHz}$, p<0.0001). From these values we estimate approximately 2% higher stiffness ($M^{\prime }\propto \nu_{B}^{2}$) and roughly 25% lower viscous loss (${M^{\prime }}^{\prime }\propto \nu_{B}\times \text{FWHM}$) in scar tissue, consistent with increased deposition of oriented collagen that replaces the normal basket‐weave ECM [56].

The FWHM values measured in this study differ from previously reported results, most likely due to methodological differences. In studies performed under mechanical loading, external forces expel interstitial fluid, reducing viscosity and amplifying stiffness changes [50, 57]. By contrast, Brillouin spectroscopy probes tissue in its native, unloaded state, capturing the intrinsic acoustic response of the local viscoelastic matrix.

This interpretation is supported by our incidental observation that a mechanically compressed region of healthy skin (pinched by labeling tape, Figure 4) displayed both increased $M^{\prime }$ and decreased ${M^{\prime }}^{\prime }$.

The FWHM values exhibit a standard deviation 50% larger than that of the scar region, reflecting intrinsic heterogeneity and hydration gradients across different dermal layers.

The conclusion regarding the presence of fibrosis in the wound region is based on pronounced morphological changes in the tissue, including a recessed appearance, discoloration, absence of hair follicles, and loss of surface texture. While it could be argued that neoplastic or inflammatory skin changes might produce similar morphological alterations, these conditions are typically associated with increased tissue volume and other characteristic features [58, 59], which were not observed in the present case.

In summary, Brillouin microspectroscopy can distinguish fibrotic dermis from healthy dermis in vivo, in a label‐free manner, by mapping stiffness and viscous loss at sub‐epidermal depths [40]. These findings highlight the potential of this approach to extend to other fibrotic tissues that share the hallmark features of dense collagen deposition, fibrillar cross‐linking, and altered fluid distribution.

The future direction of this study is to replicate the experiment in a controlled animal model. This approach would provide a larger dataset for statistical analysis and help clarify potential site‐dependent changes in skin stiffness, as well as inter‐individual variability.

## 4 Conclusion

Our results demonstrate the novel application of Brillouin spectroscopy for non‐invasive, non‐contact assessment of the mechanical properties of human dermis. This study demonstrates that Brillouin microspectroscopy is a sensitive tool for in situ, non‐destructive characterization of skin elasticity. It provides a promising method toward quantifying the rheological properties such as stiffness and viscoelasticity of dermal fibrotic regions without requiring tissue excision or causing patient discomfort.

These findings suggest that Brillouin spectroscopy could be an effective method for monitoring tissue recovery and fibrosis progression, offering potential for faster diagnosis and real‐time tracking of pathological fibrotic conditions not only in skin but potentially for other optically accessible organs.

## Conflicts of Interest

The authors declare no conflicts of interest.

## Data Availability

The data that support the findings of this study are available from the corresponding author upon reasonable request.
